# Predicting binding sites from unbound versus bound protein structures

**DOI:** 10.1038/s41598-020-72906-7

**Published:** 2020-09-28

**Authors:** Jordan J. Clark, Zachary J. Orban, Heather A. Carlson

**Affiliations:** Department of Medicinal Chemistry, College of Pharmacy, University of Michigan, Ann Arbor, 428 Church Street, Ann Arbor, MI 48109-1065 USA

**Keywords:** Computational biophysics, Molecular biophysics

## Abstract

We present the application of seven binding-site prediction algorithms to a meticulously curated dataset of ligand-bound and ligand-free crystal structures for 304 unique protein sequences (2528 crystal structures). We probe the influence of starting protein structures on the results of binding-site prediction, so the dataset contains a minimum of two ligand-bound and two ligand-free structures for each protein. We use this dataset in a brief survey of five geometry-based, one energy-based, and one machine-learning-based methods: Surfnet, Ghecom, LIGSITE_csc_, Fpocket, Depth, AutoSite, and Kalasanty. Distributions of the F scores and Matthew’s correlation coefficients for ligand-bound versus ligand-free structure performance show no statistically significant difference in structure type versus performance for most methods. Only Fpocket showed a statistically significant but low magnitude enhancement in performance for holo structures. Lastly, we found that most methods will succeed on some crystal structures and fail on others within the same protein family, despite all structures being relatively high-quality structures with low structural variation. We expected better consistency across varying protein conformations of the same sequence. Interestingly, the success or failure of a given structure cannot be predicted by quality metrics such as resolution, Cruickshank Diffraction Precision index, or unresolved residues. Cryptic sites were also examined.

## Introduction

Interactions between proteins and small molecule ligands are a cornerstone of biochemical function. These interactions vary in specificity, which allows for invention of new molecules to modulate the function of protein targets. Modern drug discovery heavily utilizes structure-based drug design, which requires structural information for the target of interest (typically a protein). As structural information for new targets is obtained, there are cases where little is known about the orthosteric and allosteric binding pocket(s) of the protein. Consequently, significant effort has been invested into the development of ligand binding-site (LBS) prediction algorithms to help solve this issue. As with many computational methodologies, extensive testing and validation of these algorithms has been a common topic of literature review^1–3^. Unfortunately, due to the timespan of these different validation and benchmarking publications, very few of them use the same dataset. Another common theme among the datasets is the underrepresentation of ligand-free (apo) crystal structures, as most datasets are disproportionately populated with ligand-bound (holo) structures, simply mirroring the relative population of the Protein Data Bank (PDB)^4^.

In this study, we address the question of how much influence a starting structure has on the resulting LBS prediction. Previous studies have been mixed on the issue. Chen et al. reported that geometry-, energy-, and consensus-based methods benefitted from the use of holo structures^5^. However, An et al. found that using apo versus holo pockets had little effect on the resulting prediction of LBS^6^. There are two aspects we focused on. First, do bound or unbound structures work better, and second, how much variability is seen in the results across many structures of the same protein. To address these questions, we had to create a new dataset.

Our current dataset is derived from our previous study of protein flexibility^7^, and it originates from Binding MOAD^8,9^, a collection of high-quality holo crystal structures. Corresponding apo structures were acquired using sequence-based searches of the PDB and similar quality assessment criterion to the curation process for Binding MOAD. Starting from the 4048 crystal structures used in the previous study, we culled this dataset to 1446 holo structures and 1082 apo structures, representing 304 unique protein families (as clustered by 100% sequence identity) where all structures have resolution of 2.5 Å or better and all holo structures contain ligands that are biologically relevant. Additionally, each of the 304 unique proteins is represented by at least two ligand-bound and at least two ligand-free structures. Unified binding sites (UBSs) have been calculated for all protein families in this dataset, which represent the union of *all* residues contacted by *any* bound ligand within a family. This dataset is much larger and contains many more ligand-free structures than any of the datasets used in past work. The curation process for this dataset is described in the “Methods” section.

Previous LBS-prediction algorithms have been trained and tested using numerous different datasets and databases. Some methods^10–12^ were trained and tested using manually curated datasets from the PDB and some^13,14^ using previously established datasets such as LigASite^15^ or the Astex diverse set^16^. Older publications were more likely to use manually curated datasets, as some of the publicly available datasets and resources were not yet available. Resources such as LigASite^15^ and BioLiP^17^ have since been created with the direct intent for use in prediction method training and testing since that time. The APoc^18^ and TOUGH-M1^19^ datasets also deserve mention in this context, though these datasets are intended for use in benchmarking binding-site similarity methods and thus contain sequence-dissimilar protein pairs.

Cimermancic and coworkers created the CryptoSite dataset of 84 binding sites annotated as being “cryptic”^20^. Cryptic sites require structural rearrangement for ligands to access to the binding pocket. When analyzing these cryptic pockets using Fpocket^21^ or ConCavity^13^, the apo structures yielded pocket scores much lower than their holo counterparts. Proteins containing cryptic binding sites are of significant interest to the biochemical community, and they represent particularly difficult cases for binding-site detection. However, characterizing these pockets using only two methods leaves room for the application of many additional methods.

The recently published CavBench^22^ benchmarking tool is highly related to the work herein. Its CavDataset is a curated subset of PDBsum^23^, consisting of 2293 protein structures with 660 apo and 1633 holo structures. This dataset is larger than many of the previous datasets of its kind, but it only shares ~ 5% (122 structures) overlap with the dataset used in our work here. Additionally, the number of sequence-related or sequence-unique proteins contained in the CavDataset is not specified by the authors. The authors contrast the performance of four prediction methods (Fpocket^21^, GuassianFinder^24^, Ghecom^25^, and KVFinder^26^) on their CavDataset based on different classifications of pockets, as well as the apo or holo nature of the starting structures. The performance of all four methods appears completely unaffected by the presence or absence of ligands in the starting structure.

Given the previous findings, we see a need for (1) a more robust dataset for benchmarking computational prediction methodology using multiple conformations of the same protein and (2) further investigation into the implications of ligand presence on starting structure performance.

### LBS-prediction methods

LBS-prediction methods are divided into four categories for discussion: template-based methods (sometimes referred to as genomic-based methods), geometry-based methods, energy-based methods, and other methods.

Template-based methods utilize the atlas of already known protein information as a map to guide the algorithm. Their assumption is that binding sites of new protein sequences may be located using the known binding sites of close structural homologs. Some examples of template-based methods include: 3DLigandSite^27^, FINDSITE^28^, Firestar^29^, I-TASSER^30^, IntFOLD^31^, and ProBis^32^.

Geometry-based methods characterize protein surfaces using observable biophysical parameters such as Van der Waals radii in order to locate pockets or clefts, assuming that the binding site of the protein is one of these pockets or clefts. Exploration of the protein surface may be accomplished by calculation of molecular distance, solvent accessible surface area, and cavity volume. These measurements may be computed using probes, spheres, grids, and other forms of spatial voids, which are then clustered or further analyzed to yield ranked cavities presumed to be binding sites. Geometric methods have the advantage of not requiring any prior knowledge about a protein target or any of its close structural relatives, aside from having structural information to work with. This property is advantageous to the purpose of this work. Some examples of geometry-based methods include: SURFNET^10^, Ghecom^25^, LIGSITE_csc_^11^, Fpocket^21^, Depth^12^, and the CAVITATOR algorithm as part of APoc^18^.

Energy-based methods rely on calculation of phenomena such as hydrogen bonding and pi-stacking to locate regions of the protein where ligands are likely to bind. These methods utilize probe molecules and chemical moieties to generate potentials for locating binding sites. Some examples of energy-based methods include: AutoSite^14,33^, PocketFinder^6^, SiteHound^34^, Q-SiteFinder^35^, and FTSite^36^.

The idea of sequence conservation has been incorporated into other methods, such as the upgrade from LIGSITE to LIGSITE_csc_ by including a re-ranking of top predicted pockets using sequence conservation^11^. Propensity-based methods rank potential binding pockets on a by-atom basis (in the context of likelihood of interacting with a bound ligand) and tally up scores of predicted pockets to either rank novel pockets (e.g. LISE^37,38^) or re-rank pockets from other methods (e.g. STP algorithm^39^, Hirayama’s method^40^).

Machine-learning methods have been the primary focus of many recent developments, which incorporate any number of previously established physicochemical parameters into a machine-learning context. These methods utilize computational prediction algorithms ranging from relatively simple Random Forest decision trees to sophisticated neural networks trained on dozens of physicochemical parameters. An example is Gutteridge and Thornton’s neural network method^41^, which predicts the likelihood of a residue being catalytic in nature, where neural network inputs consist of: solvent accessibility, type of secondary structure, depth, cleft the residue resides in, as well as conservation score and residue type. LIBRUS^42^ is a support vector machine learner which primarily utilizes sequence-based information, but performed poorly. Similarly, LigandRFs^43^ utilizes random forest ensembles to predict binding sites purely from sequence information. Though it was one of the best sequence-based performers, the authors note that structure-based methods still outperform sequence-based ones, even with the help of machine learning^43^. Some of the newest developments in this area include DeepSite^44^, DeepCSeqSite^45^, Kalasanty^46^, and UTProt Galaxy pipeline^47^. These methods all utilize 3D convolutional neural networks with various information involving sequence, distances, and other physicochemical parameters to characterize putative binding pockets.

There are also newer methods deemed as ‘meta-analyses’ which combine multiple methods and multiple types of methodology, with some variety of a re-scoring algorithm to try and achieve the best facets of each type of method they use. Some examples include ConCavity^13^ which uses 3 methods and MetaPocket^48^ which uses 8 methods. While robust, these methods are only available as webservers and cannot be seamlessly integrated into many method pipelines. Also, users have limited options to customize parameters for their purposes.

Template-based methods are typically among the best performers during large-scale benchmarking exercises such as the contact prediction section of the Critical Assessment of Protein Structure Prediction (CASP)^49^. The “LBS-prediction” section and its separate assessment in CASP has not appeared since CASP round X^50,51^. The Continuous Automated Model EvaluatiOn (CAMEO) webserver where users may test their server-based automated methods is also of importance for benchmarking^52^. Template-based methods have been excluded from our comparisons, as utilizing libraries of sequence-based template information would inevitably lead to the use of holo structure knowledge to solve the binding site locations in apo structures.

For this work, we elected to use seven methods: SURFNET, Ghecom, LIGSITE_csc_, Fpocket Depth, AutoSite, and Kalasanty. The first five of the methods are geometry-based, while AutoSite is energy-based, and Kalasanty is machine-learning-based. Choosing methods was based on two primary factors. First, the availability of source-code to be installed and used in house was required, as using web-servers for large amounts of data was not a viable option. For instance, the source code for CAVITATOR^18^ was not readily available, and the source code for DeepCSeqSite^45^ was a limited demo at the time of this work’s completion. Secondly, we excluded methods that had shown poor performance in previous benchmarks, as detecting performance differences between different structures of the same target is further complicated when the methods are not performing well in general. Our choice of methods is by no means exhaustive and is simply intended to provide a set of base information as to how LBS-prediction methods perform on different types of structures.

## Results and discussion

### Dataset properties

The most recent release of Binding MOAD^8^ was clustered using a very strict sequence identity cutoff to obtain relevant holo structures, and matching apo structures were obtained from the PDB as described in the “Methods” section. Upon filtering for proteins with at least two holo structures and two apo structures and reducing all families to a maximum of ten structures for each apo/holo state (see “Methods” section), this dataset reduces to 304 different protein families, represented by 1446 holo structures and 1082 apo structures. This dataset is available online as the file LBSp_dataset.tar.gz at https://BindingMOAD.org/Home/download.

The protein families with the most holo structures prior to family size reduction are carbonic anhydrase II followed by trypsin, with 174 and 120 holo structures, respectively. The protein families with the most apo structures before size reduction are lysozyme followed by ribonuclease-A, which had 280 and 79 apo structures, respectively. This redundancy is accounted for in two major ways. First, when describing prediction assessment for each protein family, the value will be given as an average, median, maximum, or minimum for the entire family as one value to represent all contained structures. Second, families with more than 10 of either type of structure are reduced to the 10 most diverse (via RMSD) representatives for prediction calculations. For example, the carbonic anhydrase II family has 174 holo structures, and all of the ligands for the 174 structures are used to build the UBS, so all structures are truly represented; however, only the 10 most diverse holo structures are used in the prediction calculations to save computational time. This process is detailed in the “Methods” section. The results of this family size reduction are 2528 protein structures (1446 holo, 1082 apo) which are actually tested with every one of the seven LBS-prediction methods. This data is provided as part of the Supplementary Information; Table S1 lists the results of each LBS-prediction method on each of the structures in our dataset.

**Figure 1:**
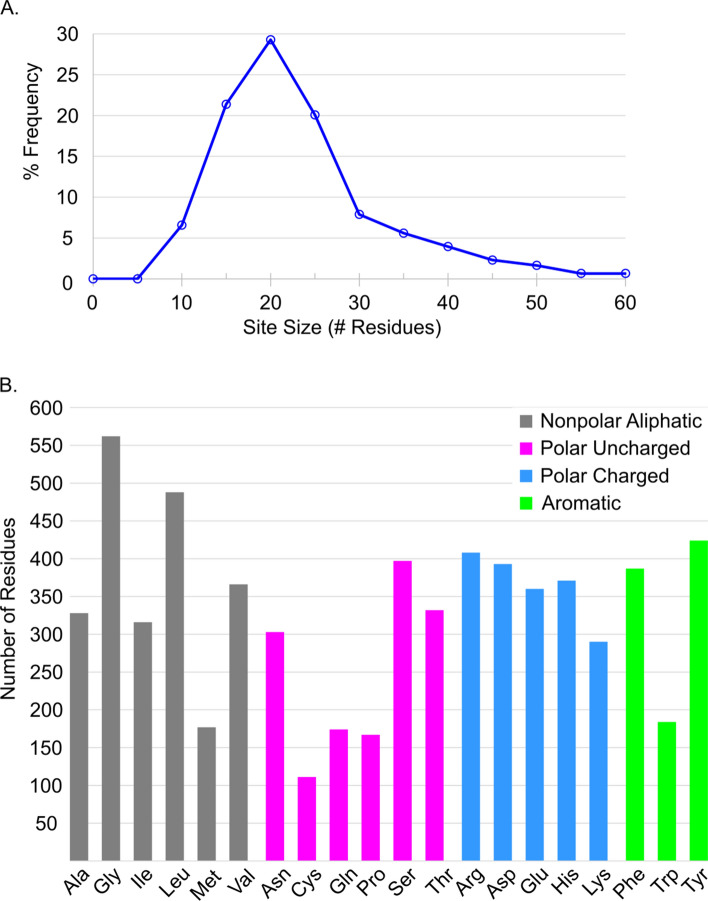
(**A**) Distribution of the sizes of unified binding sites for the 304 protein families in this dataset, as % frequency. (**B**) Distribution of amino acid composition of the 304 unified binding sites.

The biologically relevant ligands that occupy the holo structures in this dataset are diverse and represent many different classes of molecules. The average molecular weight (MW) of the ligands is 374 g/mol with 80% of ligands less than 500 g/mol and 95% less than 800 g/mol. This large range in molecular size helps with building diverse UBSs. The distribution of UBS sizes and number of each residue type represented across all binding sites are presented in Fig. 1a and b.

**Figure 2:**
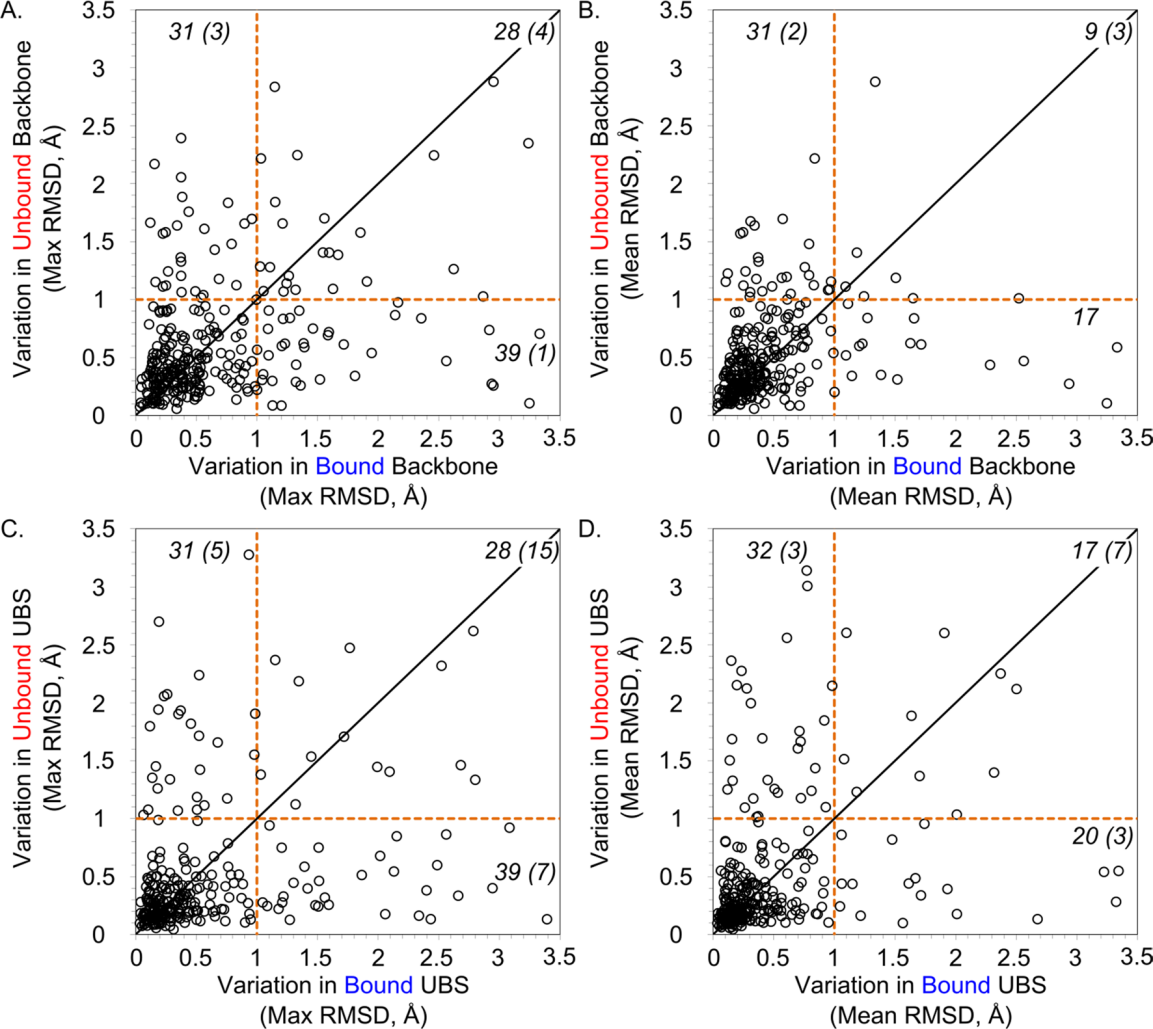
Analyses of maximum and mean backbone RMSD for each protein family. Each point represents the maximum or mean observed in one protein family, and the number of points of each section is labeled in black (numbers in parenthesis are points with values > 3.5 Å). (**A**) The maximum backbone RMSD across the apo-apo pairs is compared to the maximum of the holo-holo pairs; 206 proteins display RMSD ≤ 1 Å for both groups. (**B**) The mean backbone RMSD across the apo-apo pairs is compared to the mean of the holo-holo pairs; 247 proteins display RMSD ≤ 1 Å for both groups. (**C**) The maximum UBS RMSD across the apo-apo pairs is compared to the maximum of the holo-holo pairs; 206 proteins display RMSD ≤ 1 Å for both groups. (**D**) The mean UBS RMSD across the apo-apo pairs is compared to the mean of the holo-holo pairs; 235 proteins display RMSD ≤ 1 Å for both groups.

The proteins and their binding sites are fairly rigid as a whole dataset, in terms of C_α_ RMSD. Exhaustive C_α_ RMSD calculations were completed for all 304 protein families, for both the global backbone and specifically for those residues characterizing the UBS of the family. The maximum and average RMSDs for all 304 protein families are presented in Fig. 2. In the most focused-case (Fig. 2c), 68% of the protein families display negligible amounts of backbone motion throughout their UBS.

### LBS prediction

Predictive power is assessed using two metrics in this work: F scores and Matthew’s Correlation Coefficients (MCCs). Justification for this type of analysis and description thereof can be found in “Methods” section. Comparison between performance of different methods, or of different sets of data (apo vs. holo), will be represented by *p* values from Wilcoxon rank-sum tests. The Wilcoxon rank-sum (also known as the Mann–Whitney test) was chosen because it requires no prior knowledge of the shape of the distribution nor its symmetry about the mean or median. The F scores and MCCs provide a good description of the relative success of these algorithms, but the absolute case of failure is based on whether or not a method produced a predicted binding site containing *none* of the residues in common with our definition of the UBS. This is not to say that only accounting for at least one residue in a given site should be commended, but rather that predicting one correct residue or more implies the algorithm is close to the “correct” location on the binding surface of the protein.

Here, we interject that our analysis is based on the classical approach of using *p* < 0.05 to denote statistically significance between sets or distributions of data. This approach has been called into question in our community^53^, and so we provide all *p* values in full instead of truncating them at the 0.05 threshold. While investigating beyond that 0.05 threshold holds the temptation of meaningful information, we instead strive to gain additional insight by having a large and robust dataset. Representing all 304 unique proteins in multiple structures of both the bound and unbound forms will yield a more robust approach than many past studies which have adhered to apo-holo protein pairs.

Biounit files for all 2528 protein structures were prepared as described in the “Methods” section. All structures were submitted to each of the seven LBS-prediction methods and the top predicted pocket of each method was analyzed. For the methods that yield a grid representation of the binding site rather than actual binding-site residues (AutoSite, LIGSITE_csc_), the binding sites were back-calculated using a distance cutoff of 4.5 Å unless a different cutoff was specified in the citation for the method (8 Å for LIGSITE_csc_^11^). Any structures which did not yield any predicted pockets were assigned zero values for MCC, precision (P), recall (R, also referred to as sensitivity or true-positive rate), and F score, after they were inspected to ensure the programs completed their calculations properly. The procedure for dealing with structures that resulted in errors for the various methods, as well as a list of these very few structures, is provided in the “Methods” section. Analysis metrics were then calculated for the rest of the resulting structures using in-house parsing scripts.

### Apo versus holo structures

**Figure 3:**
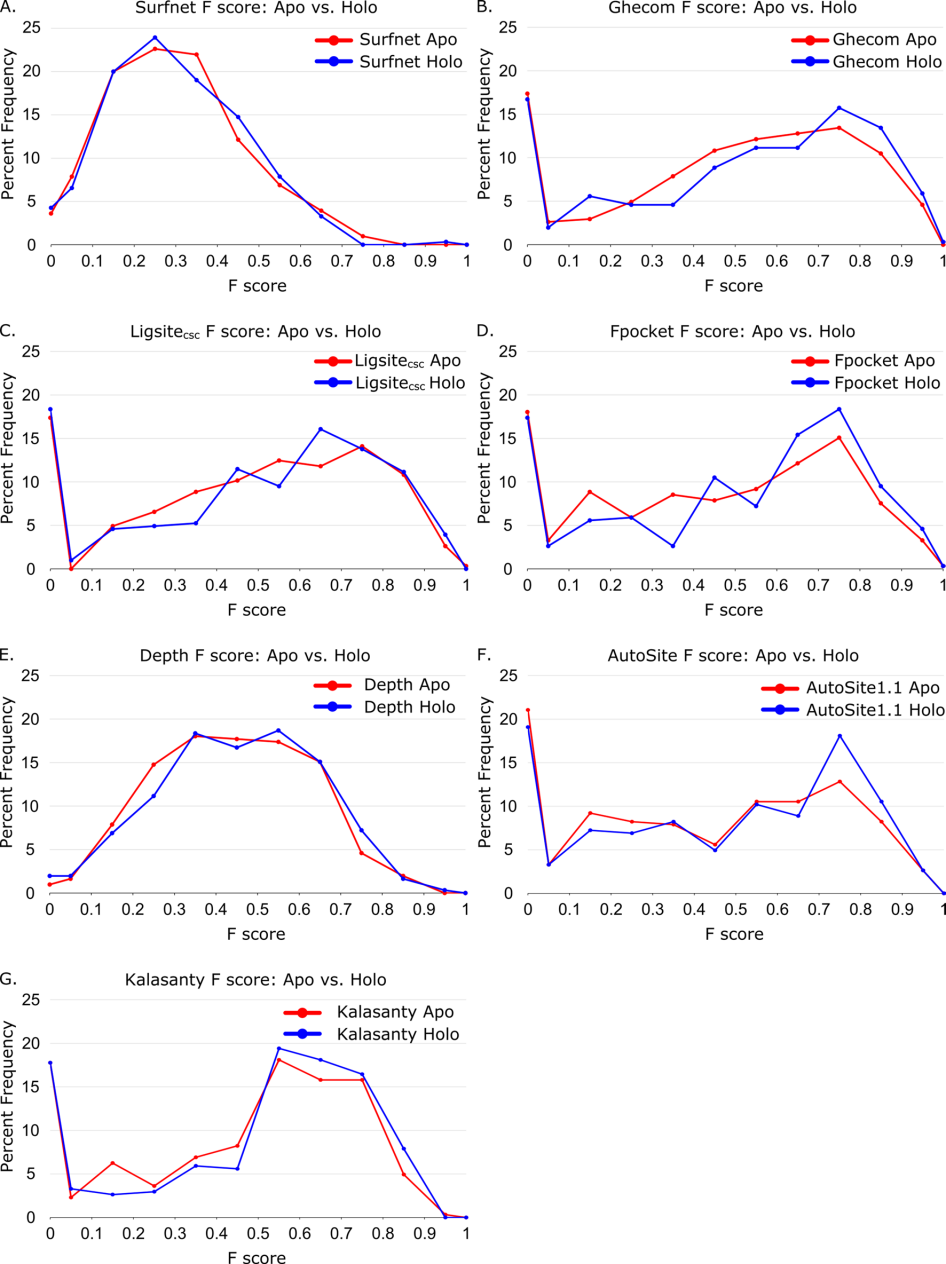
Distribution of family median F scores of apo and holo protein structures for (**A**) Surfnet (*p* = 0.90), (**B**) Ghecom (*p* = 0.20), (**C**) LIGSITE_csc_ (*p* = 0.56), (**D**) Fpocket (*p* = 0.04), (**E**) Depth (*p* = 0.32), (**F**) AutoSite (*p* = 0.13), and (**G**) Kalasanty (*p* = 0.12).

Our analysis of the predictive power for the seven LBS-prediction methods begins with presenting distributions of F scores for all methods (Table 1, Fig. 3) These are distributions of the family median F scores, divided into the subcategories of apo structures and holo structures. Though it is interesting that the holo structures have higher F scores across most methods, it is important to note that the Wilcoxon rank-sum analysis of apo versus holo distributions yields *p* > 0.05 for all methods except for Fpocket (*p* = 0.04). We note that the large populations with F scores of zero for Ghecom, LIGSITE_csc_, Fpocket and AutoSite are structures that either do not predict the correct binding site as their #1 predicted site or structures where no site is predicted at all (a rare occurrence, see “Methods” section). Additionally, we note that these figures are of the family median values. At first glance, Ghecom, LIGSITE_csc_, Fpocket, AutoSite, and Kalasanty all appear to have similar rates of zero-score occurrences (17–20% of the data). However, many of these families have an average F score > 0, indicating that there is success in at least one of the structures within a given family.

**Table 1:** Median of family median F scores and MCCs for apo and holo datasets for all seven LBS-prediction methods.

Method	Apo F	Holo F	Wilcoxon *p*: F score	Apo MCC	Holo MCC	Wilcoxon *p*: MCC
Surfnet	0.23	0.23	0.90	0.22	0.23	0.63
Ghecom	0.48	0.54	0.20	0.50	0.53	0.17
LIGSITE_csc_	0.49	0.52	0.56	0.47	0.50	0.60
Fpocket	0.42	0.53	**0.04**	0.43	0.52	**0.03**
Depth	0.40	0.42	0.32	0.38	0.40	0.17
AutoSite	0.36	0.45	0.13	0.34	0.42	0.10
Kalasanty	0.49	0.51	0.12	0.48	0.54	0.11

**Figure 4:**
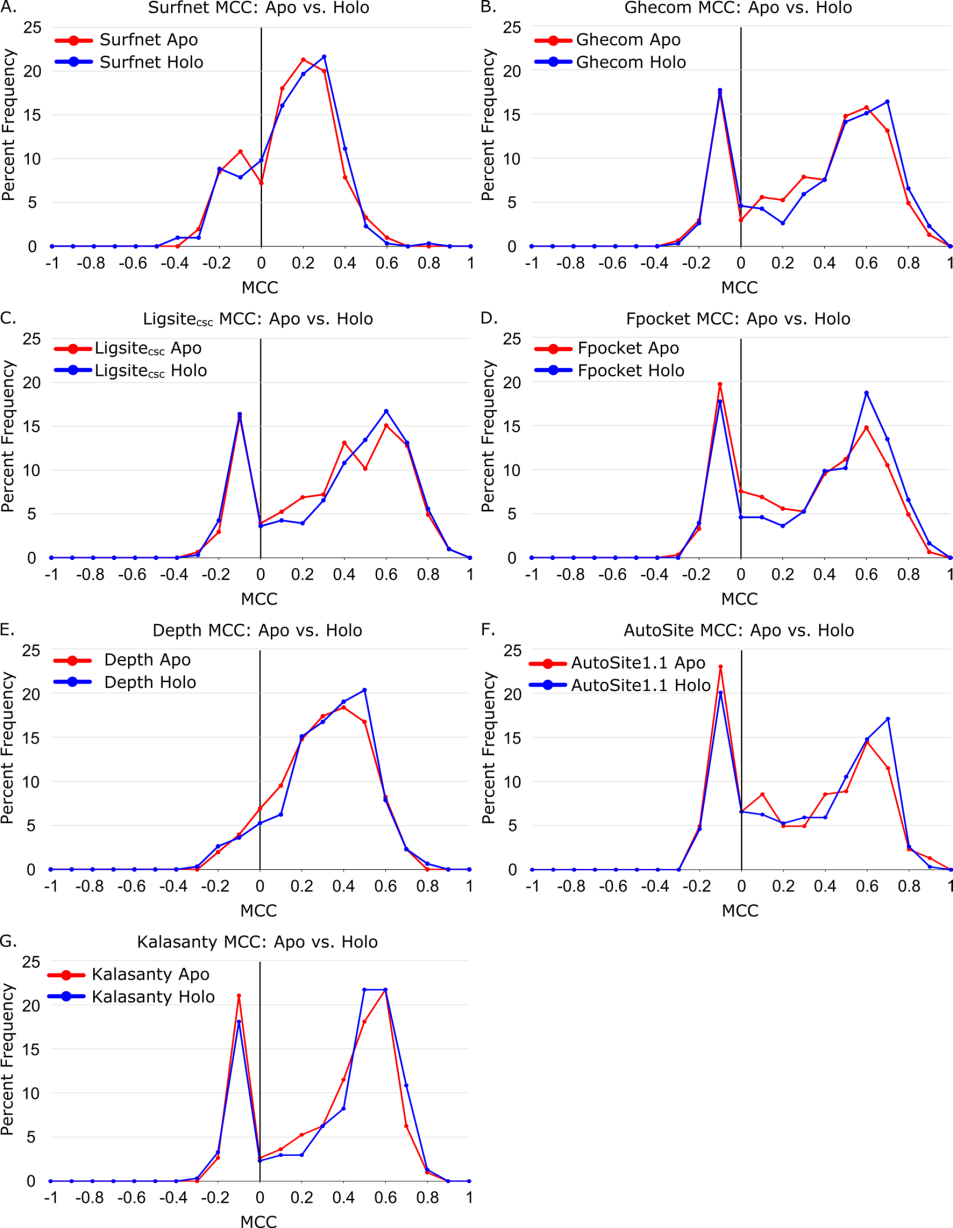
Distribution of family median Matthews Correlation Coefficients (MCCs) of apo and holo protein structures for (**A**) Surfnet (*p* = 0.63), (**B**) Ghecom (*p* = 0.17), (**C**) LIGSITE_csc_ (*p* = 0.60), (**D**) Fpocket (*p* = 0.03), (**E**) Depth (*p* = 0.17), (**F**) AutoSite (*p* = 0.10), and (**G**) Kalasanty (*p* = 0.11).

Wilcoxon *p* values are the same as those found in Figs. 3 and 4.

The bold values are the only ones that meet the statistical limit of *p* < 0.05.

While Fpocket does appear to have a slight performance preference for holo protein structures, the other six methods show no statistically distinguishable preference for apo or holo structures. This implies that the predictive power for most of these methods is not heavily impacted by the presence or absence of a pre-organized binding site with a ligand in the starting structure. The same trend is observed when using MCCs as the evaluation metric of predictive power (Table 1, Fig. 4). Only Fpocket (*p* = 0.03) has a statistically significant (*p* < 0.05) correlation between predictive power and structure type (holo vs. apo), again suggesting that holo structures perform slightly better with this method, but the trend is weak. CavBench’s assessment showed equal performance of Apo and Holo protein structures in non-redundant binding site detection for Fpocket and for Ghecom^22^.

For both MCC analysis and F scores, two primary patterns of predictive power are observed. Surfnet and Depth appear to have a higher likelihood of mid-level predictive power (F score < 0.7, MCC < 0.6), while also having a much lower rate of complete failure (zero-scores). The other five methods appear to have a more bimodal distribution of scores, either accurately predicting a relatively large portion of the binding site or failing completely in their top predicted site (18–22% of protein families).

### Variability across protein structures

Perhaps the wildly varying performance of the different methods could be attributed to specific structures or proteins. To probe this idea, the failed prediction cases were assessed more closely. Of the 2528 protein structures (1446 holo, 1082 apo) processed with these methods, only six structures failed catastrophically (zero correct binding site residue predictions, R = 0) in every one of the seven methods. Given the size of the whole dataset, that ~ 0.2% failure rate is very low. There were 1215 structures for which at least one method failed to predict any part of the binding site (R = 0). However, 974 of those 1215 structures have at least 50% of their binding site predicted (R > 0.5) by at least one other method. The performance of many structures appears to be dissimilar between the methods. Exhaustive comparison of the resulting F scores and MCCs for each individual PDB structure between every combination of the seven LBS-prediction methods was performed, resulting in correlation R^2^ < 0.1 for every comparison. This suggests that the performance of any structure with one method provides no indication of how that structure will perform with another method. This variability (most PDB structures doing very well with one method, yet failing with another) compromises the analyses of how to improve LBS-prediction methods in general.

**Figure 5:**
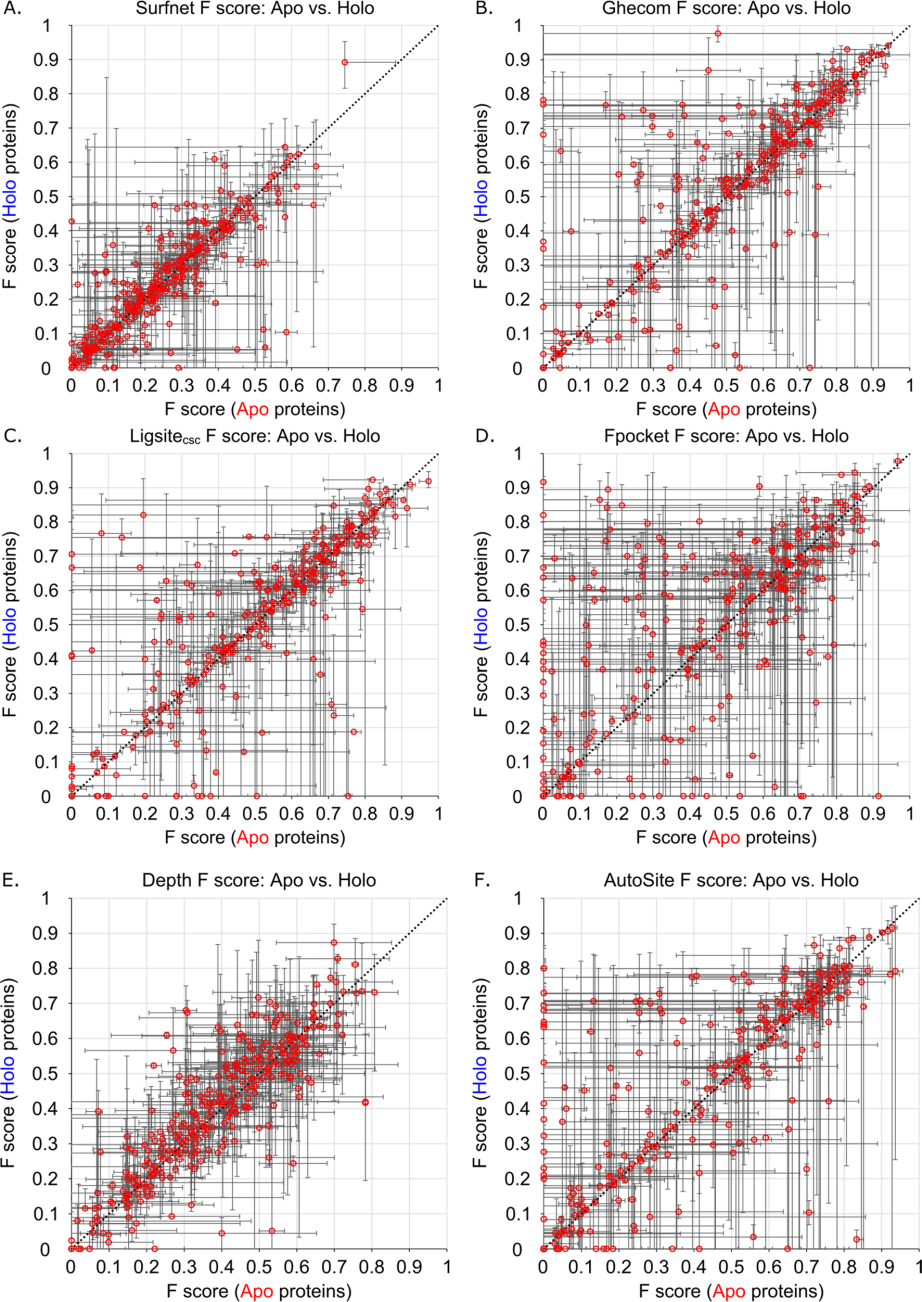

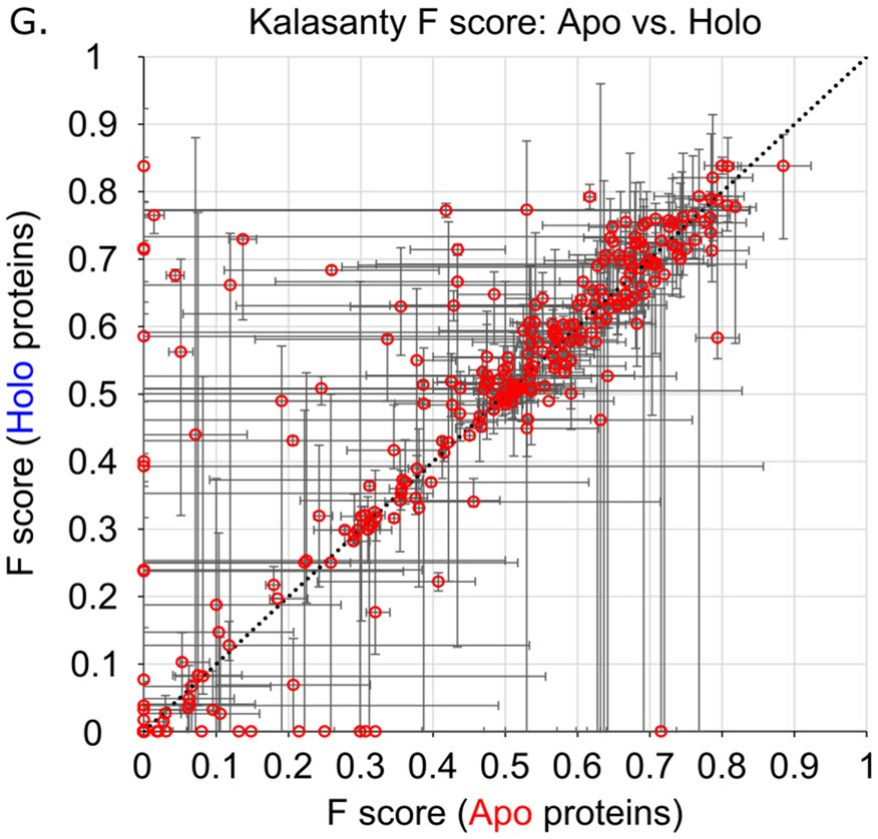
Family median F scores of apo and holo protein structures for (**A**) Surfnet, (**B**) Ghecom, (**C**) LIGSITE_csc_, (**D**) Fpocket, (**E**) Depth, (**F**) AutoSite, and (**G**) Kalasanty where the error bars are constructed from the family minima and maxima. Line: *y* = *x.*

**Figure 6:**
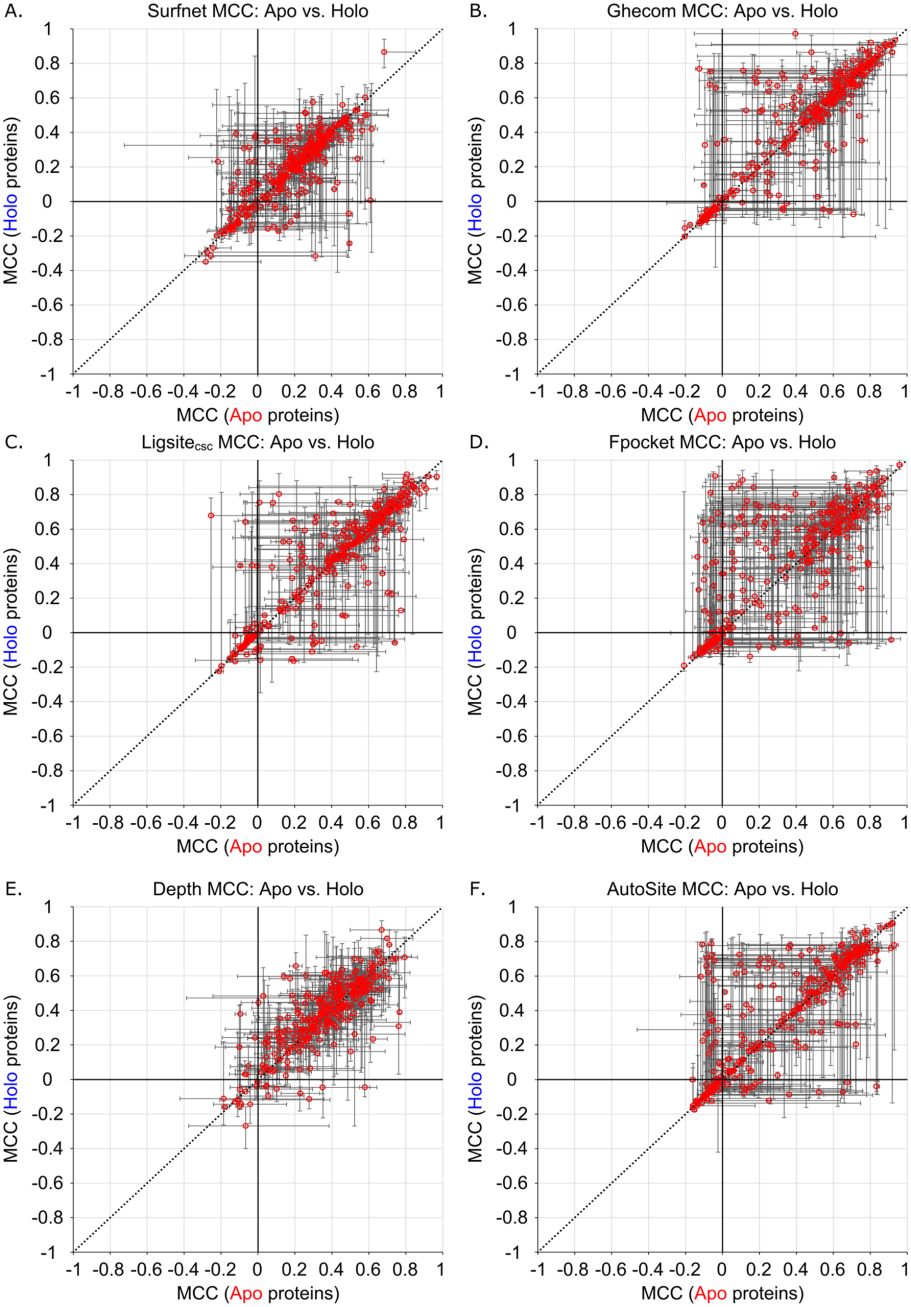

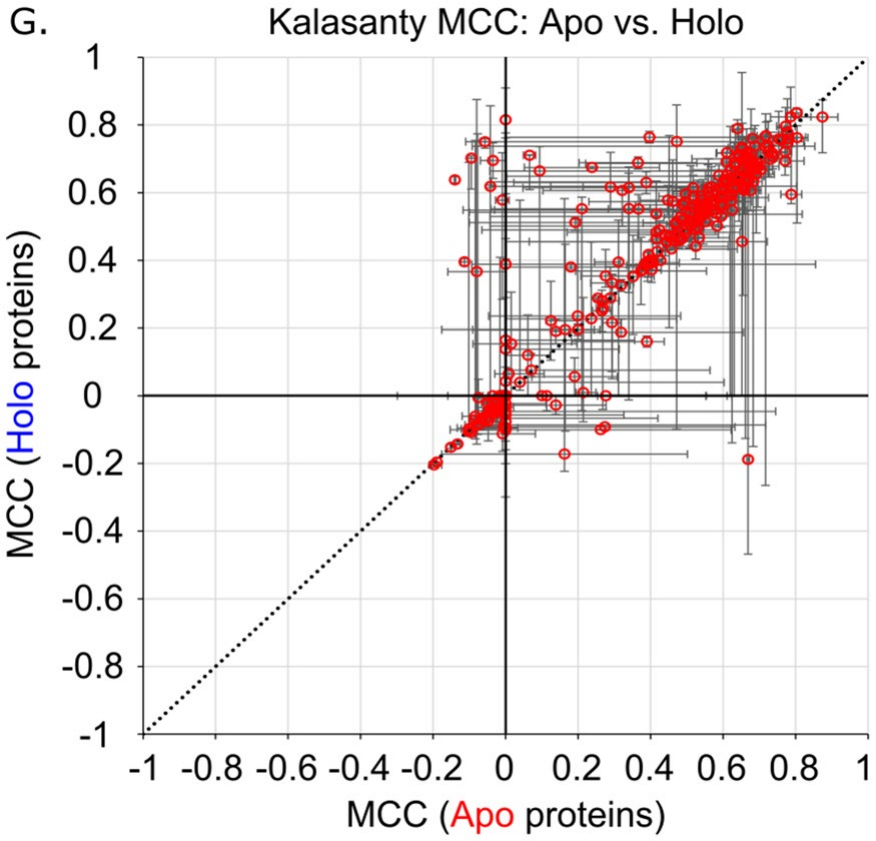
Family median MCCs of apo and holo protein structures for (**A**) Surfnet, (**B**) Ghecom, (**C**) LIGSITE_csc_, (**D**) Fpocket, (**E**) Depth, (**F**) AutoSite, and (**G**) Kalasanty where the error bars are constructed from the family minima and maxima. Line: *y* = *x.*

. Another analysis for the success of each method is to view the F-scores and MCCs as a by-family comparison between the two structure types (i.e. how do the apo structures of a given protein perform relative to the holo structures of the exact same protein?). Using family medians for the representative family data points, and family minima/maxima as error bars, the predictive power of the seven methods is presented for the F scores in Fig. 5 and MCCs in Fig. 6. Interestingly, family maxima and minima span the gamut of performance for each method for nearly all of the 304 protein families in both F scores and MCCs. This is to say: for most of the protein families in this study, there are structures for which each method will accurately predict the majority of the ligand-binding site, as well as structures where the same method completely fails to identify any portion of the same binding site as the top predicted site. This observation is true for both the apo and holo states of the proteins and has serious implications for benchmarking LBS-prediction methods, as the choice of protein structures greatly influences outcome. This inherent variability makes it impossible to rank methods and points to a need for greater consistency on the part of the methods, as well as community effort towards more robust and commonly utilized benchmarking datasets.

### Cryptic sites

The authors of CryptoSite^20^ annotated 84 examples of known cryptic binding sites. Cryptic binding sites require notable protein rearrangement to become apparent, embodying the concepts of induced-fit and conformational selection. Extending this idea to the computational paradigm, cryptic sites are not as easily identifiable by pocket detection algorithms when using their apo structures, according to the authors. Our dataset in this work shares 30 of the same PDB structures (13 holo, 17 apo) with the CryptoSite set. Moreover, 35 of the 304 protein families in our dataset are represented in the CryptoSite set, as determined by sequence identity and inspection (see “Methods” section). In 31 of those 35 families, we are investigating the same “cryptic” binding site as CryptoSite, as determined by < 0.5 Å distance between bound ligands between our holo structures and the ligand contained in the designated holo structure from CryptoSite.

. The performance of those 31 families when compared to the remaining 273 families in our dataset was nearly identical, overall. Assessing the difference in distribution of family median performance scores for the 31 overlap families vs the remaining 273 families yielded *p* values > 0.1 for both F score and MCC, for both the apo protein values and holo protein values. The CryptoSite authors defined accurate predictions of cryptic sites to require sensitivity (recall) values of > 0.33. Using that threshold, 99.3% and 99.7% of our apo and holo protein structures, respectively, have successful predictions from at least one method.

Though we did not observe statistically different performance of cryptic binding pockets compared to the rest of our dataset, we have annotated the overlap with the CryptoSite set accordingly. The full dataset download for this work is appropriately separated, and the annotations of its contents (Supplementary Information Tables S1 and S2) are also labelled accordingly.

### Relationships between method performance and structural data

Was it possible that the performance of a given crystal structure in any LBS-prediction method was related to the overall quality of that structure? Structure quality was assessed in two ways: structure resolution and Cruickshank Diffraction Precision Index (DPI)^54^. There is some redundancy here, as resolution is used in the calculation of DPI, but DPI is a far more complete measure of X-Ray crystal structure quality. Across all comparisons of F score versus resolution, F score versus DPI, MCC versus resolution, and MCC versus DPI, the highest correlation R^2^ value obtained was 0.03. This implies no correlation between structure quality and the performance of the structures in any of the LBS-prediction methods showcased here.

As an additional metric of quality, unresolved residues were considered. For these experiments, the UBS were examined across all structures within a family, and any missing (unresolved) residues were noted. Residues outside of the binding sites were not tallied in this process. There were 61 families in the dataset which had at least one structure with at least one UBS residue missing. The performance of the structures in those families were compared on a per-family basis, i.e. the structures without any missing residues versus the structures with at least one residue missing. Structure type (apo or holo) was not considered for this analysis. Of the 61 families with missing residues, only 19 showed any statistically significant difference in performance.

If unresolved residues were problematic in this analysis, their impact would likely appear in the performance metrics of every method we tested. Instead, 12 of the 19 families only showed statistically significant performance differences for one method, and they were not always the same method: AutoSite (3 families), Surfnet (1 family), Depth (3 families), Ligsite_csc_ (1 family), Ghecom (4 families). Fpocket and Kalasanty did not show any differences for performance in any family. Of the remaining 7 family cases, 6 of the families showed differences with only two methods, and the last case showed significant differences with five of the seven methods.

Most interestingly, the impact of these structures with missing residues is not always negative. The family of Concanavalin A, which showed statistically significant performance differences for six methods (the most of any of these families), has three apo structures (1apn, 1dq2, 1enq) which are missing some residues in the binding site and seven apo structures without any missing residues, as well as 10 holo structures which are not missing any residues. The performance of the five methods (Surfnet, Ghecom, Ligsite, Depth, and AutoSite) improves on the structures which have missing residues, in every case.

Structures missing UBS residues were uncommon, and those structures causing any significant difference on the performance of any of the methods were exceedingly less common still. As such, we elected to not exclude any of these data, as missing residues appear to have an overall miniscule impact on the performance of the methods.

Relationships between method performance (F scores/MCC) and other structural features were examined. Protein family Min/Max/Average/Median F and MCC values for every method were compared to family Min/Max/Average/Median global C_α_ RMSD, maximum family UBS C_α_ RMSD, UBS size, Cruickshank DPI, and resolution for both apo and holo proteins. The resulting 896 comparisons yielded R^2^ values between 0 and 0.03, showing no correlation for any method with any physical property of either the apo or holo proteins. As an example, Figs. S1 and S2 in the Supplementary Information show the comparisons between family maximum RMSD vs family median F score and MCC score, respectively, which show no relationship between the full extent of protein flexibility and performance for any of the LBS-prediction methods.

## Conclusions

The predictive power of the seven LBS-prediction algorithms did not appear to correlate with the ligand-bound state (apo vs. holo) of the protein structure being used. This implies that, contrary to historical belief, apo structures can perform as well as, or better than, holo structures. Previous studies have been mixed on the issue of apo versus holo structures^5,6,22^, and the behavior described here is attributed specifically to our dataset. Our complete data for this manuscript is provided in the Supplementary Information covering all calculated P, R, F, and MCC value for all individual PDB structures (Table S1 in the Supplementary Information) and giving median MCC and median F for the whole protein families (Table S2). In order to extend this idea to other computational methodology, more high-quality datasets need to be made available to the community which have proper representation of apo structures.

Our UBS definition may be deemed too generous, and it may aid methods that tend to “over-predict” binding sites, which is a somewhat expected problem with LBS-prediction methods. This is mostly due to the nature of binary classification, where many false positives are extracted when a model is pushed towards 100% recall rate. The top-10 LBS-prediction methods in round IX of CASP^55^ had an average MCC of 0.62 for the 129 targets in that round. While we cannot directly compare to this value, as the datasets and methods being tested are not similar, it does give a reference value for what state-of-the-art methods are capable of in competition. Due to this, the lack of F scores and MCCs with values close to 1 is unsurprising. The average MCC of our methods was 0.38 for Apo structures and 0.44 for Holo structures, both of which are below what we initially expected for the average performance of these methods.

In closing, none of the 304 binding sites that we have characterized in this work appear to be cryptic binding pockets under the definitions put forth by Cimermancic and coworkers^20^. Of the 1082 apo structures in our dataset, only two had no effective predictions across all of the seven prediction methods. Those two structures both belonged to families where the other apo structures had successful predictions with at least one of the seven methods.

## Methods

### Dataset construction

The dataset used in this study is available as the file LBSp_dataset.tar.gz online at https://BindingMOAD.org/Home/download. Holo structures were derived from Binding MOAD^8^, a source of high quality protein–ligand complexes that have a maximum of 2.5 Å resolution. Biologically relevant ligands are differentiated from opportunistic binders (e.g. salts, buffers, phosphate ions) in the crystal structures of Binding MOAD, making curation of relevant ligand structures straightforward. Furthermore, use of Binding MOAD excludes covalently bound ligands. Structures with more than one biologically relevant ligand were excluded from this study in favor of binary protein–ligand complexes to ensure that only one pocket was being analyzed in each protein. Any structures containing additional molecules in their binding site, such as additives, were also excluded.

Holo structures were then clustered by 100% sequence identity in both directions, without replacement (to ensure a non-redundant dataset). A subsequent 95% sequence identity clustering of those families was then performed to suggest any families that should be merged due to simple N or C terminal amino acid additions. Sequence identity between structures was determined using BLAST^56^. Any families differing in protein core sequence were kept separate.

Apo structures were then cultivated from the PDB using the same bidirectional 100% sequence identity BLAST procedure, requiring better than 2.5 Å resolution^57^. Structures were screened for bound molecules, and only those containing acceptable additives or no additives at all were kept. Acceptable additives were restricted to HET groups of 5 atoms or less and a MW of 100 Daltons or less. Each HET group was inspected for chemical appropriateness.

Finally, proteins that did not have at least two holo structures and two apo structures were excluded from the dataset at this point.

### Family size reduction

After construction of the UBS (described below), but before binding-site prediction, protein families with more than 10 structures of a single type (apo, holo) were reduced to 10 of those type of structures utilizing the following procedure: Exhaustive pairwise RMSDs were calculated for each family (every possible apo-apo, apo-holo, and holo-holo combination) using Gaussian weighted RMSD methodology developed previously in our laboratory^58^. Matrices were constructed for holo-holo pairs, and separately apo-apo pairs, for families in need of reduction. These matrices were then utilized in PAM clustering (Partitioning Around Medoids) in the R statistical package to determine the 10 most diverse structures to represent a family at hand^59^. For example, the largest family (family 1) of Lysozyme had to be reduced from 280 apo structures to 10 apo structures (utilizing a 280 × 280 pairwise RMSD matrix).

Theoretically, because the entirety of the holo structure set is used to construct the UBS prior to this data reduction, their influence on the outcome of the experiment remains. This reduction was only intended to reduce computation time for the prediction methods. These methods reduced the dataset from 2369 holo and 1679 apo structures, to 1448 holo and 1026 apo structures. Lastly, due to poor binding site resolution in structure 1HNK, which resulted in having 10 binding site residues unresolved, its entire family (two apo, two holo) was removed from the dataset. This results in the final dataset of 304 protein families, with 1446 holo and 1082 apo structures.

### Dataset overlaps

Kalasanty was trained using the majority of the sc-PDB^60^. Without an explicit list of which structures were used, we made the assumption that any structure contained in the sc-PDB was used in their training. For this overlap, we simply considered which PDB IDs from our dataset of 2528 structures existed within sc-PDB. The result was 395 structures, representing 135 of our 304 sequence-unique protein families.

By direct PDB ID comparison, we shared 30 structures (13 holo, 17 apo) with CryptoSite. We also opted to find matching sequences between our dataset and CryptoSite. This was accomplished using BLAST^56^ with a sequence identity cutoff of 90% between our 304 protein families and all of the 186 PDB structures listed within CryptoSite. After visual inspection of an RMSD alignment, 35 total sequences were deemed to be the same protein (≥ 90% sequence identity) in each of the pairings. However, only 31 of our protein families were represented in the CryptoSite dataset. The binding sites being investigated in the remaining 4 families were different in our dataset than those identified as cryptic in the CryptoSite set.

### File choice, setup, and preparation

These steps were taken prior to any binding-site calculations. The first biounit model containing the relevant ligand of the corresponding PDB structure was used by default for each structure. Only one copy of the appropriate binding chain(s) was kept in order to prevent mapping the same sites multiple times across many multimeric copies of the proteins. All hydrogens were removed from the files. All ligands and waters were removed from the files.

All protein systems were renumbered utilizing the pdbSWS database prior to binding site calculation and assembly^61^. In the cases where this would result in more than one numbering pattern inside of a family, one structure’s numbering was applied to the other structures. If this was not possible and there was no method to renumber a structure to the same pattern as the rest of its family apart from manual processing, it was discarded from the dataset out of consideration for reproducibility.

Renumbering structures was necessary because some structures were numbered differently (especially common when going between apo and holo structures). Protein numbering becomes critically important in the case of UBSs, where it is necessary to harvest residue data from the UBS when there are no ligands present to define the site (apo structures).

After binding sites were identified (detailed in the following section), the files were reduced to contain only the chain(s) involved with a single copy of a binding site.

### Binding site identification and compilation of the “union” binding site (UBS)

The binding site was defined to include all protein residues within 4.5 Å of any biologically relevant ligand for each protein, which should capture both hydrogen-bonding and van der Waals interactions. Hydrogen atoms were not considered during this 4.5 Å calculation (for either the protein or the ligand). Most of the crystal structures for a given protein had different ligands bound, so many could have a slightly different set of residues near the ligand. Therefore, the summation of all sets of residues in all complexes for each protein was used to identify the “union” binding pocket for that protein, i.e., unified binding site (UBS).

### Responding to computational errors

Structures which resulted in errors when submitted to a particular method were very uncommon, and most of the time reformatting the PDB file in some manner alleviated the issues (eg. the multiple residue conformation issue detailed in the AutoSite section below). Structures which produced errors for the various methods are provided below in Table 2. Notably, none of these structures were problematic with more than one method. Importantly, failures of 3n5k and 1su4 with Surfnet occur due to the algorithm attempting to generate an interaction array which is larger than a hard-coded threshold value. We opted to not edit the source code to fix this error.

**Table 2:**
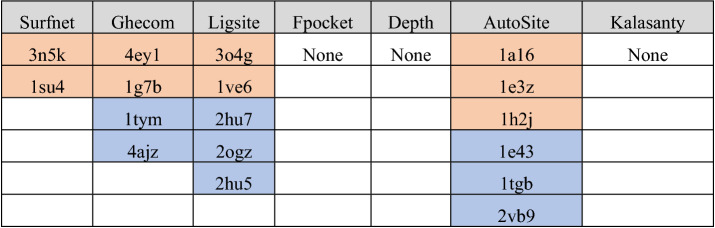
PDBids for structures which resulted in system errors for the various LBS-prediction methods.

Apo structures are denoted in orange, holo structures are denoted in blue.

### Responding to empty prediction files

For all but two methods (Fpocket and Kalasanty), structures yielding no predicted pockets were an extremely rare occurrence (Table 3). Fpocket yielded no predicted pockets for 40 different structures (17 apo, 23 holo). Kalasanty yielded no predicted pockets for 198 different structures (93 apo, 105 holo). Yielding zero pockets resulted in a score of zero for precision, recall, F score, and MCC. These failures were double checked by-hand as single command-line submissions, to ensure no other issues were taking place.

**Table 3:** PDBids for structures which resulted in no predicted pockets for the various LBS-prediction methods.

Method (data)	Structures with no pockets
Ghecom (Apo)	1g7b,4ey1
Ghecom (Holo)	1tym,4ajz
Ligsite (Apo)	1ve6,3o4g
Ligsite (Holo)	2hu5,2hu7,2ogz
Fpocket (Apo)	1aki,1b2d,1g7b,1guj,1mi7,1rnu,1u1t,1uoj,1yy6,1zz6,2rh2,2vjz,3a93,3az5,3w3b,4bwo,4f4t
Fpocket (Holo)	1a7x,1b0d,1j4h,1our,1tym,1uzv,1zt9,2boj,2oly,2olz,2z3h,3dcq,3ipe,3qe8,4ajx,4ajz,4b4q,4b4r,4joj,4jor,4lkd,4tun,4tz8
Depth (Holo)	2olz
AutoSite (Apo)	1b2d,1n40,1vie,2vjz
AutoSite (Holo)	1uof,1vif,2oly,2rk2,3lb2,4ajz
Kalasanty (Apo)	1alv,1b2d,1bmz,1dq2,1ed8,1f41,1fz2,1fz7,1fz8,1g7b,1gmq,1gwg,1hfj,1ier,1ird,1l7l,1m47,1mi7,1mmi,1mso,1n1z,1nxd,1ous,1oux,1pw9,1r13,1r14,1r7i,1sar,1tta,1u6j,1u94,1uoj,1w6l,1w8e,1yy6,1yze,2ajs,2cm3,2duo,2g4g,2gqv,2gt7,2i3u,2i4e,2j46,2noy,2pol,2ptx,2rh2,2vjz,2wlc,2wld,2×88,2yf3,2yf4,2yf9,3a4d,3c95,3d5g,3d7p,3e8m,3enr,3exx,3f32,3gxm,3kv7,3kx7,3o7s,3par,3q4j,3q6e,3rnt,3ssw,3vaf,3vag,3vaj,3wne,4b4p,4bwo,4clf,4ey1,4f4t,4i2g,4j0c,4k3s,4lse,4lsf,4lsh, 4ovh,4usv,8rnt,9rnt
Kalasanty (Holo)	1alw,1eta,1ew8,1ew9,1fy5,1gic,1gmr,1i3h,1m49,1n20,1n22,1ona,1ovs,1rnt,1rsn,1tym,1uzv,1wav,1wpg,1wrp,1xgi,1xms,1xvd,1xz3,1yvx,1zt9,2ajz,2boj,2bp6,2duq,2dur,2flm,2foj,2foo,2fop,2oly,2olz,2omg,2omi,2oz9,2r1x,2r1y,2r2b,2rk2,2roy,2sar,2wle,2wlf,2wlg,2wos,2yfd,2ys6,3bpc,3d1f,3d1g,3d5i,3dcq,3dh2,3eio,3f33,3f34,3f35,3f37,3f38,3hl8,3ikn,3ikp,3ikq,3ikr,3imu,3iqf,3kw1,3paq,3qce,3qcf,3sy0,3t4y,3vq5,3vq8,3vqe,4ajx,4ajz,4akj,4b4q,4b4r,4bu4,4gcq,4hjt,4i87,4j0i,4k3m,4k3r,4l6o,4lk7,4lkd,4lke,4lkf,4mjq,4mjr,4n94,4n97,4n9a,4usu,5cna,6rnt

### Assessment metrics

Statistical tests (correlation R^2^, t-tests, distribution analysis, and Wilcoxon rank-sum where appropriate) were completed using the software package JMP^62^. Receiver Operator Characteristic (ROC) curves are a classic method for analysis of these types of data. However, it has been thoroughly discussed that analyzing ROC curves for performance of functional residue prediction can be highly misleading^42,63^.

We have therefore chosen to assess methods utilizing metrics intended for binary classification events revolving around the four elements of the resulting confusion matrix: True Positives (TP), False Positives (FP), True Negatives (TN), and False Negatives (FN).

The scoring of each algorithm’s site prediction follows: Any residue predicted to be part of the binding site which was present in our UBS definition was denoted TP. Any residue predicted to be part of the binding site which was *not* in our UBS definition was denoted FP. Any remaining residues in the USB definition not accounted for in an algorithm’s predicted binding site were denoted FN. All remaining residues (which were not predicted as part of the binding site and not part of our UBS definition) were denoted as TN. The total number of residues present in each individual structure was taken into account for these calculations, as different structures of the same protein will commonly have different numbers of terminal residues resolved.

Both precision (P) and recall (R) analyses, as well as Matthew’s Correlation Coefficients (MCCs), have proven to be useful in the assessment of prediction methods^13,25,64^. As such, we use MCCs, as well as F scores, which are calculated from precision and recall (Eqs. 1–4), as metrics to represent the predictive power of the various methods.

1 $$MCC = \frac{{\left( {TP*TN} \right) - \left( {FP*FN} \right)}}{{\sqrt {\left( {TP + FP} \right)\left( {TP + FN} \right)\left( {TN + FP} \right)\left( {TN + FN} \right)} }}$$

2 $$P = \frac{TP}{{TP + FP}}$$

3 $$R = \frac{TP}{{TP + FN}}$$

4 $$F = 2*\frac{P*R}{{P + R}}$$

Precision is value between 0 and 1 where 1 is a perfect score, representing the likelihood of a method’s predictions to be correct. Recall is a value between 0 and 1 where 1 is a perfect score, representing what percentage of the true correct answer is represented by the true positives predicted by the algorithm. The F score is a value that represents the harmonic mean of precision and recall. In our analysis, F score values were presented instead of P or R values because they simplified the predictive power of a method into a single number for easier comparison between different methods or data types (holo vs. apo).

Precision and recall do not account for true negatives in any way and are thus blind to the relative ratio of possible answers that could be derived; in this case, this alludes to the size of the binding site in relation to the size of the protein. While going as far as rewarding methods for correct true negatives (metrics such as accuracy) would be disadvantageous to the purpose of this work, MCCs are a good medium where correct true negatives are not rewarded, and false positives are still punished albeit less severely. The MCC also has the advantage of being one stand-alone metric, where precision and recall need to be condensed to an F score to provide a single figure. Both P/R and MCCs have been used in LBS-prediction benchmarks in the past. We will present both F scores (representing P and R) as well as MCCs for a more robust analysis.

### Prediction method parameters

Depth is a geometric method based on the relationship between solvent accessible surface area and molecular depth^12,64^. Molecular depth is defined as the distance between a molecule (as an average of the distances of all of its constituent atoms) and bulk solvent. In the case of binding-site prediction, this is the depth of amino acid residues in the protein sequence from the bulk solvent outside of the globular protein. The unique characteristic of this method is its iterative solvation/resolvation technique used to determine what solvent is bulk solvent before the molecular depth calculations.

Depth was run with these conditions set in the parameters file: detection threshold of 0.8, cavity size of 4.2, resolvation cycles set to 5, solvent shell size of 4.2 Å, 25 depth cycles, minimum number of required solvent neighbors set to 4, ASA resolution of 92, ASA probe radius of 1.4 Å, and USE_MSA set to 1. A web version of DEPTH, as well as a download mirror for the software can be found at: https://cospi.iiserpune.ac.in/depth/htdocs/intro.html.

Fpocket^21^ is a pocket detection method based on alpha spheres and Voronoi tessellation^21^. Alpha spheres are spheres that contact four atoms and do not contain any atoms. Alpha spheres are constructed from the atom coordinates after calculation of Voronoi vertices, atomic neighbors and vertex neighbors. Two size-thresholds are used for classification and removal of the alpha spheres before an additional polarity classifier is assigned. The characterized alpha spheres are then subjected to a number of different clustering steps to receive putative pockets which are then ranked using the size and polarity classifiers of their contained alpha spheres.

Fpocket settings were left at their default values for pocket detection. The defaults are: minimum alpha-sphere radius (3 Å), maximum alpha-sphere radius (6 Å), minimum apolar neighbors for apolar consideration (3), minimum a-sphere per pocket (30), maximum first cluster distance (1.73 Å), maximum distance for single linkage clustering (2.5 Å), minimum number of neighbors close to each other (3), maximum distance between two pockets’ barycenter (4.5 Å), minimum proportion of apolar spheres in a pocket (0), number of Monte-Carlo iterations for the calculation of pocket volume (2500). Information about Fpocket, as well as a download mirror, can be found at: https://fpocket.sourceforge.net/.

Ghecom (grid-based HECOMi finder) utilizes spherical solvent probes in a new way^25^. The Van der Waals surface of a protein is mapped using solvent probes of varying radii. Utilizing the resulting grid information from the differently sized probes, the algorithm then incorporates mathematical morphology to simplify the protein surface and volume representations by only using small probes to describe areas where larger probes cannot reach. Ghecom was shown to reproduce the Connolly volumes derived from other methods.

Ghecom was run with large probes (mode = ‘P’), and the top binding site was defined as cluster #1 in the output clustered PDB file. The web version of Ghecom, as well as a download mirror for the software can be found at: https://strcomp.protein.osaka-u.ac.jp/ghecom/.

LIGSITE_csc_ is an update of the original LIGSITE method^11,65^. The LIGSITE_csc_ algorithm is a grid-based method where the protein is represented by its Connolly surface. The algorithm then scans for surface-solvent-surface (SSS) events and classifies solvent points which participate in greater than a user-specified threshold of SSS events as part of a pocket. These pocket-classified solvent points are then clustered to obtain putative binding pockets. If enabled, the final algorithm feature then re-ranks the three largest pockets according to residue conservation scores derived from the ConSurf-HSSP database^66^.

LIGSITE_csc_ was run with the number of pockets set to 1 (−n 1), using ‘−i’ to direct to input files through a wrapping script, with the rest of the parameters set to their default values (1 Å grid space, SSS event threshold set to 6, surface density 0.5). As pockets are provided as a centroid atom of the surface cluster, residues within an 8 Å sphere were back-calculated to represent the binding pocket. This protocol is derived from that of the authors^11^. A web version of LIGSITE_csc_ and a download mirror can be found at: https://projects.biotec.tu-dresden.de/pocket/.

SURFNET is one of the earliest LBS-prediction methods, published in 1995^10,67^. The algorithm grows spheres along the protein surface such that they reach a size where they touch two atoms on their edges and contain no other atoms. Overlapping spheres as well as spheres with a radius smaller than a user-established threshold are then discarded, and the remaining spheres are clustered into cavities. The resulting clustered cavity with the largest volume is assumed to be the putative binding site.

Gap files were generated with the following parameters (N, N, Y, 4.5) for: **N**one map-format, SITE records **N**ot required for mask region, and **Y**es to requiring neighboring atoms for mask region with a **4.5** Å cutoff (the same cutoff used for defining the binding sites from the original ligands). Binding site residues were then extracted from the generated gap files for each structure. SURFNET’s web portal can be found at https://www.ebi.ac.uk/thornton-srv/software/SURFNET/.

AutoSite^14,33^ is an energy-based method which uses AutoDock^68^ affinity maps computed with AutoGrid4^69^ for three generic atom-type grids to identify binding sites^14^. These maps are regularly spaced grids, and the three AutoDock generic atom types are hydrophobic (carbon, C), hydrogen-bond acceptor (oxygen, OA), and hydrogen-bond donor (hydrogen, HD). The computed affinity maps yield information about the sum of all interaction energies between each grid point and all receptor atoms in its local area. The algorithm then merges the three sets of high affinity points into a composite map by selecting the minimum value at each grid position. The resulting points are then clustered to find putative binding sites. Recent improvements help to merge closely spaced clusters into larger pockets^33^.

AutoSite requires PDBQT format files, which are a proprietary file format for the AutoDock suite of tools. Before generating PDBQT files for the dataset, scripts were run to remove any multiple-occupancy resides from the initial biounit files (eg. ASER, BSER, where the two occupancies would sum to 1). The highest occupancy representation for each residue was kept. This process was necessary because the PDBQT file conversion process does not accommodate multiple occupancy residues well, and results in ATOM section lines with > 80 characters that are unreadable by any PDB parser.

The PDBQT files were then generated using Autodock 1.0 Tools. During the course of this project, AutoSite was updated. AutoSite 1.1 was run with default settings, and the top predicted binding site cluster was analyzed for each protein structure (XXXX_cl_1.pdb). Actual predicted binding site residues were back-calculated from these point clusters using a 4.5Å distance cutoff. The version of AutoSite 1.1 used here is part of AutoDockFR, which can be found at https://adfr.scripps.edu/AutoDockFR/downloads.html A guide for preparing PDBQT files can be found at: https://autodock.scripps.edu/faqs-help/how-to/how-to-prepare-a-receptor-file-for-autodock4.

Kalasanty is a machine-learning method which utilizes a 3D fully convolutional neural network which characterizes protein binding pockets using physicochemical characteristics of protein atoms distributed across a 70 Å cubic grid^46^. The feature information used in their calculation describes: atom type, hybridization, number of bonds with other heavy atoms, number of bonds with other hetero atoms, encoding properties (hydrophobic, aromatic, acceptor, donor, and ring) of groups, and whether an atom belongs to a ligand or protein. These features are condensed into an 18-bit vectors for every grid cell of a 70 Å cubic grid with 2 Å spacing which is centered onto a protein. Kalasanty was trained on the majority of the sc-PDB^60^. The contents of the sc-PDB overlap with 395 structures across 135 protein families in our dataset.

Kalasanty requires Mol2 format files for input, which were generated from our PDB files using OpenBabel^70^. Their predict.py script was used to process all of our data. As this is a pre-trained machine-learning method, there are no parameters to change.

The prepublication version of Kalasanty was acquired from its Gitlab repository on 11/19/2019. The Git repository can be found here: https://gitlab.com/cheminfIBB/kalasanty.

## Supplementary information

Supplementary Information.

Supplementary Table S1.

Supplementary Table S2.

## Data Availability

The dataset of PDB structures and UBS is available from the authors as the file LBSp_dataset.tar.gz at https://BindingMOAD.org/Home/download.
