# Supplementary material for: Predicting binding sites from unbound versus bound protein structures
Source: Sci Rep. 2020 Sep 28;10:15856. doi: 10.1038/s41598-020-72906-7 (PMC7522209; doi:10.1038/s41598-020-72906-7)
Supplement: Supplementary file 1 — Supplementary Information. [file 41598_2020_72906_MOESM1_ESM.pdf]

Supplementary Information:

Predicting Binding Sites from Unbound vs. Bound Protein Structures

Jordan J. Clark, Zachary J. Orban, & Heather A. Carlson*

University of Michigan, Ann Arbor
Department of Medicinal Chemistry
College of Pharmacy, 428 Church Street, Ann Arbor MI 48109-1065

*Corresponding author email: carlsonh@umich.edu

File: Supp_Table_S1.txt
Table S1. Tab separated file of the performance of the seven LBS-prediction methods across every individual protein structure used in this analysis. The P, R, F, and MCC values for all methods are given for every individual protein in our dataset. Proteins are annotated with their PDBid, their family number, whether they are apo or holo, and whether they also appear in the structures in CryptoSite.

File: Supp_Table_S2.txt
Table S2. Tab separated file of the performance of the seven LBS-prediction methods aggregated for every protein family used in this analysis. For the apo and holo proteins in each of the 304 protein families, average RMSDs for the whole structure and just the UBS are given. Also, the median MCC and median F scores are given for each method across each protein family. It is also noted whether the protein family's sequence has ≥90% sequence identity to the structures in CryptoSite.

Figure S1. Protein flexibility across each family compared to F score. Family maximum global C_α RMSD vs Family Median F score of apo and holo protein structures for A) Surfnet, B) Ghecom, C) $LIGSITE_{csc}$, D) Fpocket, E) Depth, F) AutoSite, and G) Kalasanty.

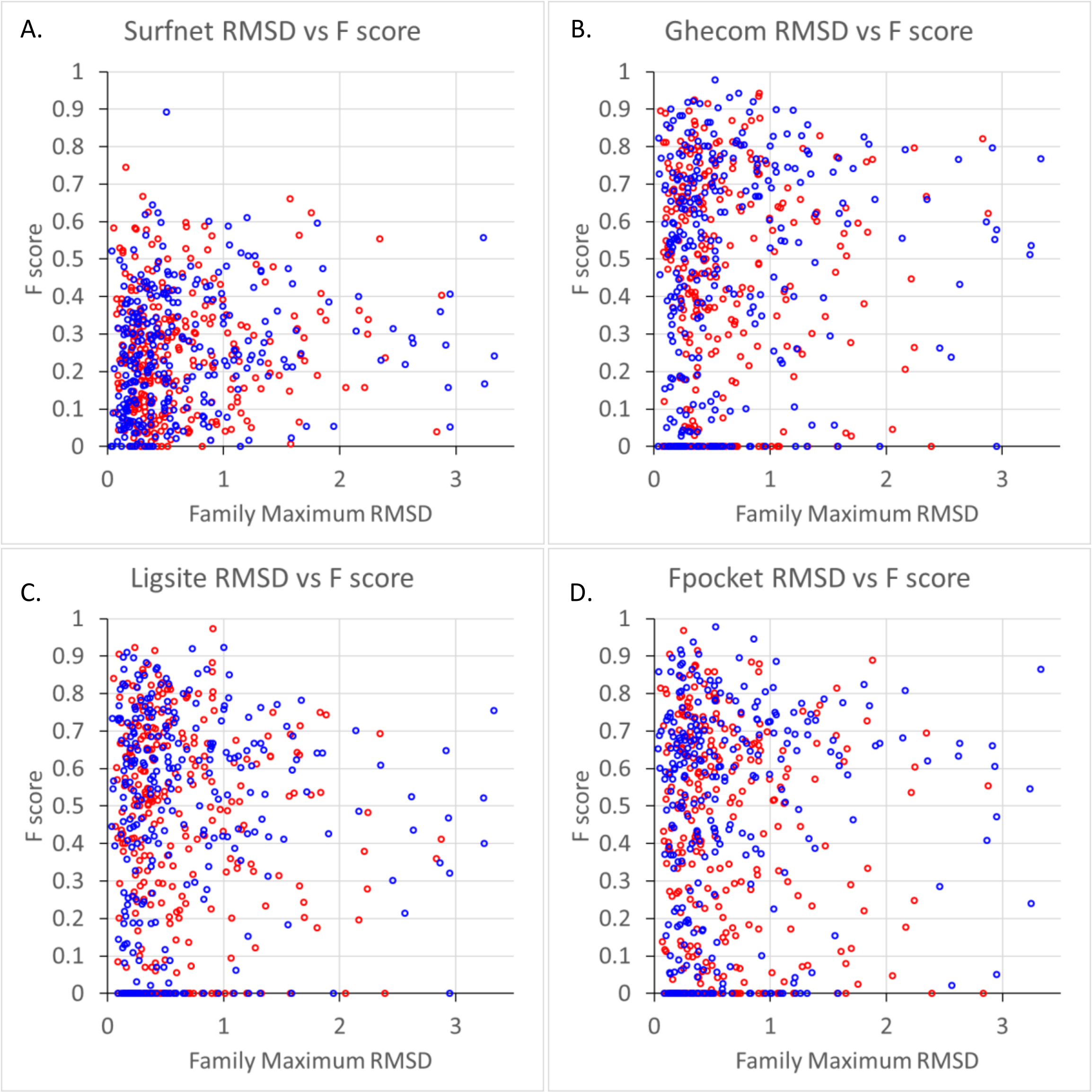

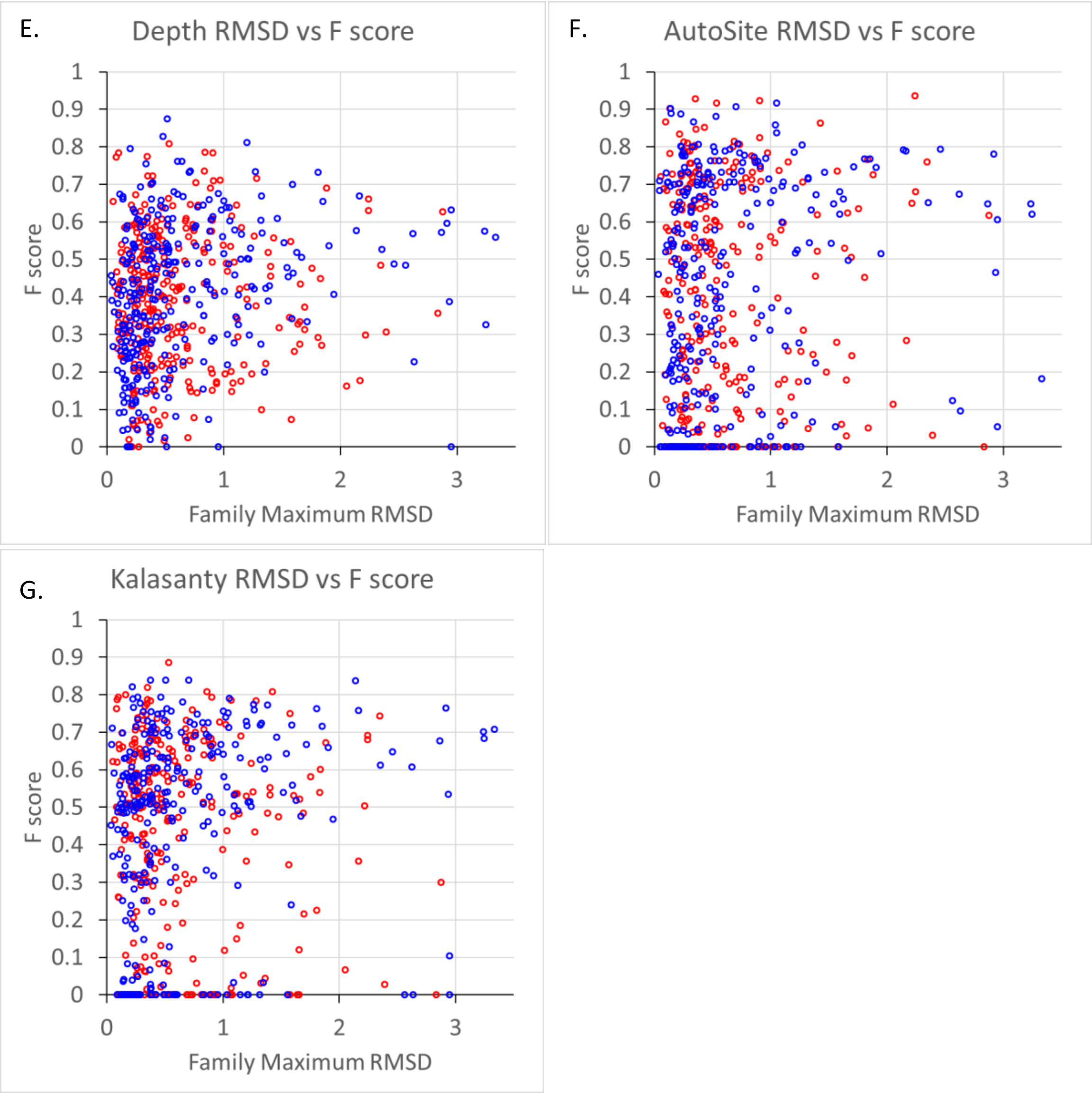
E.
Depth RMSD vs F score
F score
Family Maximum RMSD
F.
AutoSite RMSD vs F score
F score
Family Maximum RMSD
G.
Kalasanty RMSD vs F score
F score
Family Maximum RMSD

Figure S2. Protein flexibility across each family compared to MCC score. Family maximum global C_{α} RMSD vs Family Median MCC of apo and holo protein structures for A) Surfnet, B) Ghecom, C) LIGSITE$_{csc}$, D) Fpocket, E) Depth, F) AutoSite, and G) Kalasanty.

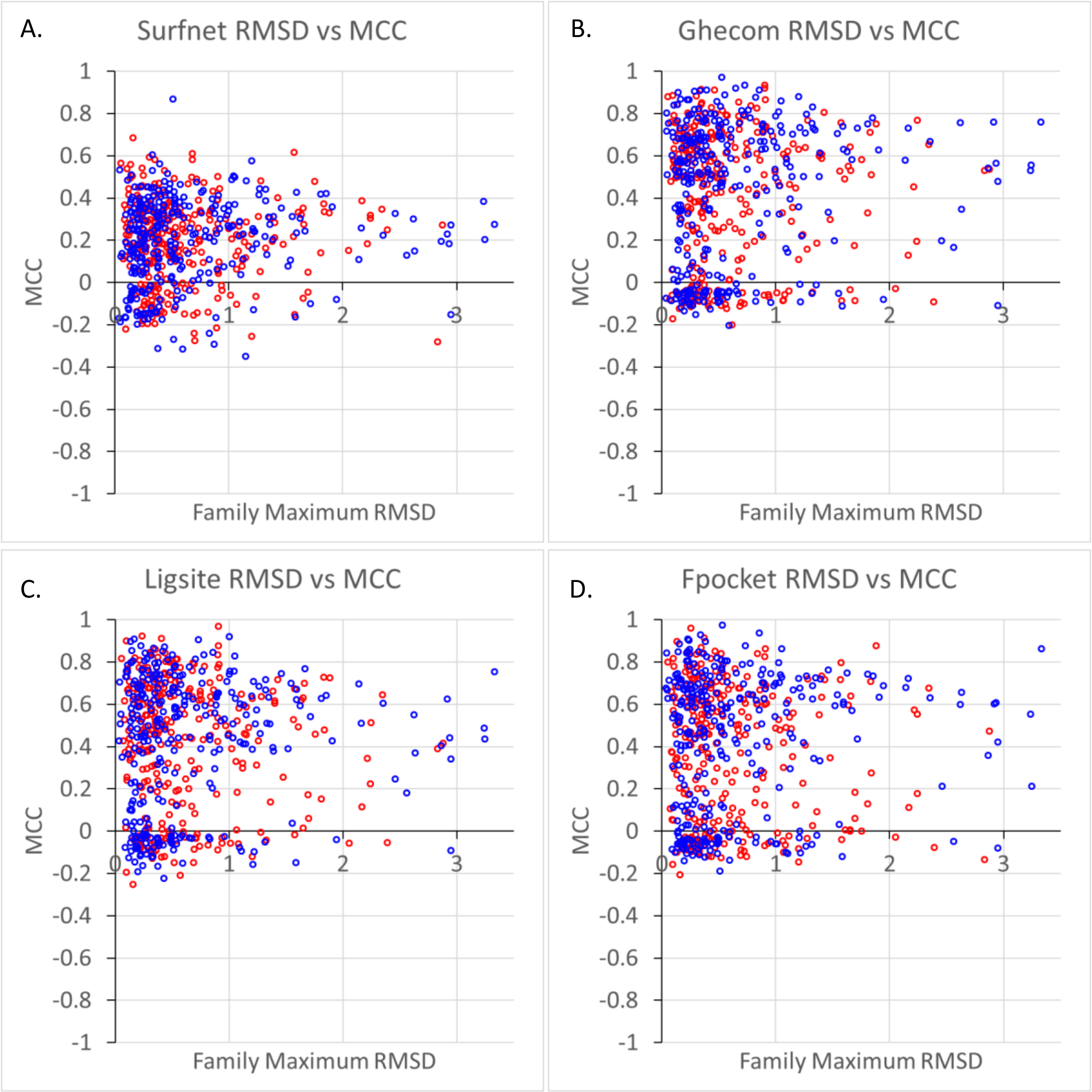

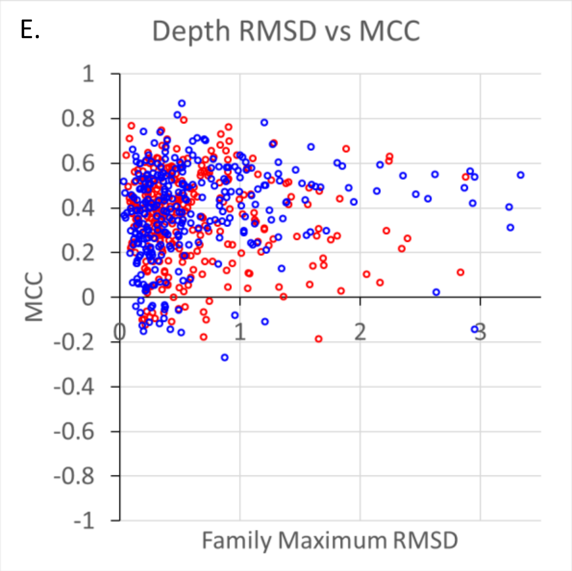
E.
Depth RMSD vs MCC
1
0.8
0.6
0.4
0.2
0
-0.2
-0.4
-0.6
-0.8
-1
MCC
0
1
2
3
Family Maximum RMSD

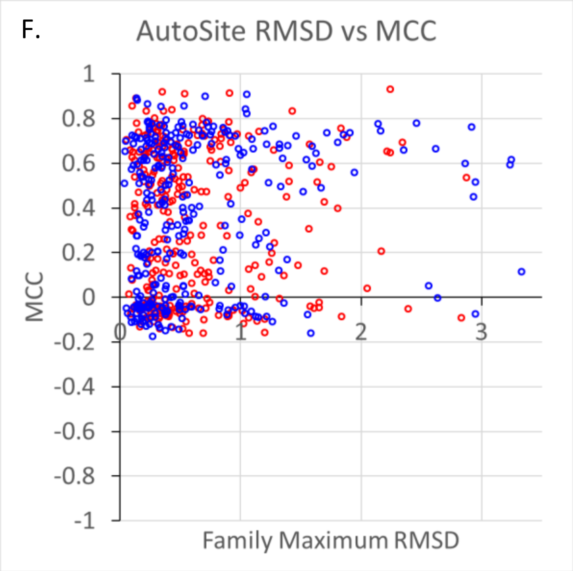
F.
AutoSite RMSD vs MCC
1
0.8
0.6
0.4
0.2
0
-0.2
-0.4
-0.6
-0.8
-1
MCC
0
1
2
3
Family Maximum RMSD

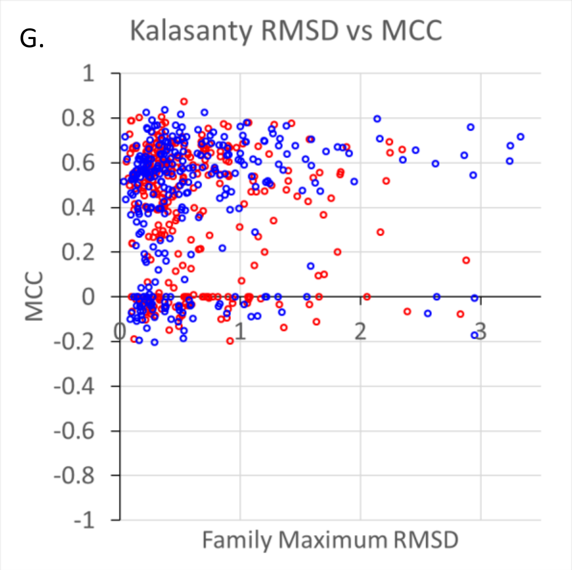
G.
Kalasanty RMSD vs MCC
1
0.8
0.6
0.4
0.2
0
-0.2
-0.4
-0.6
-0.8
-1
MCC
0
1
2
3
Family Maximum RMSD
